# Supplementary material for: Network potential identifies therapeutic miRNA cocktails in Ewing sarcoma
Source: PLoS Comput Biol. 2021 Oct 18;17(10):e1008755. doi: 10.1371/journal.pcbi.1008755 (PMC8601628; doi:10.1371/journal.pcbi.1008755)
Supplement: S1 Table — We ranked potential targets by predicted change in network potential when each protein was modeled as repressed. (PDF) [file pcbi.1008755.s004.pdf]

Supplemental Tables

	TC252	ES2	A673	TC32	EWS502	TC71
1	TRIM25	TRIM25	TRIM25	TRIM25	TRIM25	TRIM25
2	APP	APP	APP	APP	APP	APP
3	ELAVL1	ELAVL1	ELAVL1	ELAVL1	ELAVL1	ELAVL1
4	RNF4	RNF4	RNF4	RNF4	RNF4	RNF4
5	HNRNPL	HNRNPL	HNRNPL	HNRNPL	HNRNPL	HNRNPL
6	XPO1	XPO1	XPO1	XPO1	XPO1	XPO1
7	NXF1	NXF1	NXF1	NXF1	NXF1	NXF1
8	UBC	TNIP2	UBC	UBC	UBC	UBC
9	TNIP2	UBC	TNIP2	TNIP2	TNIP2	TNIP2
10	MOV10	MOV10	MOV10	MOV10	MOV10	MOV10

S1 Table. Top protein targets for each cell line. We ranked potential targets by predicted change in network potential when each protein was modeled as repressed.
