# Supplementary material for: Network potential identifies therapeutic miRNA cocktails in Ewing sarcoma
Source: PLoS Comput Biol. 2021 Oct 18;17(10):e1008755. doi: 10.1371/journal.pcbi.1008755 (PMC8601628; doi:10.1371/journal.pcbi.1008755)
Supplement: S2 Table — We ranked potential targets by predicted change in network potential when each protein was modeled as repressed, limited to proteins causally associated in cancer according to the Cosmic database. Proteins that appear in the same position for ≥ 3 cell lines are bolded. (PDF) [file pcbi.1008755.s005.pdf]

	TC252	ES2	A673	TC32	EWS502	TC71
1	**XPO1**	**XPO1**	**XPO1**	**XPO1**	**XPO1**	**XPO1**
2	**LMNA**	**LMNA**	**LMNA**	**LMNA**	NTRK1	EWSR1
3	EWSR1	**HSP90AA1**	**HSP90AA1**	EWSR1	**HSP90AA1**	LMNA
4	**HSP90AA1**	**EWSR1**	**EWSR1**	**HSP90AA1**	**EWSR1**	**HSP90AA1**
5	**CUL3**	**CUL3**	**CUL3**	**CUL3**	LMNA	**CUL3**
6	**NTRK1**	KRAS	**NTRK1**	**NTRK1**	CUL3	RECQL4
7	**TP53**	**TP53**	RECQL4	**TP53**	RECQL4	KRAS
8	RECQL4	RECQL4	KRAS	KRAS	TP53	NTRK1
9	KRAS	EGFR	TP53	RECQL4	KRAS	BRCA1
10	**BRCA1**	**BRCA1**	**BRCA1**	**BRCA1**	**BRCA1**	TP53

S2 Table. Top cancer-associated protein targets for each cell line. We ranked potential targets by predicted change in network potential when each protein was modeled as repressed, limited to proteins causally associated in cancer according to the Cosmic database. Proteins that appear in the same position for $\geq$ 3 cell lines are **bolded**.
