# Supplementary material for: Network potential identifies therapeutic miRNA cocktails in Ewing sarcoma
Source: PLoS Comput Biol. 2021 Oct 18;17(10):e1008755. doi: 10.1371/journal.pcbi.1008755 (PMC8601628; doi:10.1371/journal.pcbi.1008755)
Supplement: S3 Table — Pathways with an adjusted p-value < 0.05 are shown above. “ES” refers to enrichment score and “NES” refers to the normalized enrichment score. “nMoreExtreme” refers to the number of random gene sets (out of 500) that were more enriched than the test set. Size refers to the number of genes in the pathway that were also present in our mRNA expression dataset. (PDF) [file pcbi.1008755.s006.pdf]

	pathway	pval	padj	ES	NES	nMoreExtreme	size
1	MITOTIC_SPINDLE	0.00	0.01	0.62	1.47	0.00	197
2	DNA_REPAIR	0.00	0.01	0.59	1.37	0.00	146
3	G2M_CHECKPOINT	0.00	0.01	0.72	1.68	0.00	187
4	APOPTOSIS	0.00	0.01	0.62	1.44	0.00	158
5	PROTEIN_SECRETION	0.00	0.01	0.63	1.44	0.00	94
6	APICAL_SURFACE	0.00	0.01	0.73	1.57	0.00	42
7	UNFOLDED_PROTEIN_RESPONSE	0.00	0.01	0.62	1.43	0.00	106
8	PI3K_AKT_MTOR_SIGNALING	0.00	0.01	0.69	1.58	0.00	104
9	MTORC1_SIGNALING	0.00	0.01	0.61	1.43	0.00	193
10	E2F_TARGETS	0.00	0.01	0.72	1.69	0.00	195
11	MYC_TARGETS_V1	0.00	0.01	0.80	1.89	0.00	193
12	OXIDATIVE_PHOSPHORYLATION	0.00	0.01	0.61	1.42	0.00	184
13	ALLOGRAFT_REJECTION	0.00	0.01	0.54	1.27	0.00	191
14	MIRNA_BIOGENESIS	0.00	0.01	0.80	1.73	0.00	40
15	WNT_BETA_CATENIN_SIGNALING	0.01	0.02	0.68	1.47	2.00	42
16	ANGIOGENESIS	0.01	0.02	0.72	1.53	2.00	34
17	TGF_BETA_SIGNALING	0.01	0.03	0.65	1.43	4.00	53
18	MYC_TARGETS_V2	0.01	0.03	0.64	1.41	4.00	58
19	P53_PATHWAY	0.01	0.03	0.51	1.19	5.00	194
20	UV_RESPONSE_UP	0.01	0.04	0.53	1.23	6.00	153
21	SPERMATOGENESIS	0.02	0.04	0.54	1.24	7.00	126
22	ADIPOGENESIS	0.02	0.04	0.50	1.18	8.00	191
23	INTERFERON_GAMMA_RESPONSE	0.02	0.04	0.50	1.18	8.00	193
24	INTERFERON_ALPHA_RESPONSE	0.02	0.05	0.56	1.28	10.00	91

S3 Table. Genes ranked by network potential are enriched for several biological pathways related to cancer as well as the miRNA bio-genesis pathway Pathways with an adjusted p-value < 0.05 are shown above. “ES” refers to enrichment score and “NES” refers to the normalized enrichment score. “nMoreExtreme” refers to the number of random gene sets (out of 500) that were more enriched than the test set. Size refers to the number of genes in the pathway that were also present in our mRNA expression dataset.
