# Supplementary material for: Network potential identifies therapeutic miRNA cocktails in Ewing sarcoma
Source: PLoS Comput Biol. 2021 Oct 18;17(10):e1008755. doi: 10.1371/journal.pcbi.1008755 (PMC8601628; doi:10.1371/journal.pcbi.1008755)
Supplement: Attachment — Submitted filename: response_to_reviewers.pdf [file pcbi.1008755.s008.pdf]

Cleveland Clinic
Department of Translational Hematology and Oncology Research
9500 Euclid Avenue, Cleveland Ohio, 44195, US
July 9, 2021
e-mail: scottj10@ccf.org

Dear Dr Zhaolei Zhang,

We would like to thank all three reviewers and the editor for their assessment and feedback on our manuscript. We have updated our analysis and manuscript to address several of the concerns raised, and feel that the manuscript has been substantially improved as a result.

In addition to addressing the reviewer comments, we made substantial improvements to the code base underlying this work to enable easier reproduction or extension of our findings. We migrated the vast majority of our analytic code and processed data to a formal R package (found at https://github.com/DavisWeaver/disruptr) that can be easily installed by users, reviewers, or readers. As part of this migration, we made greater use of parallelization and C++ internals to enable calculations to be completed on much shorter time-scales than we could previously achieve. Using the R package, users can compute network potential, change in network potential, and compute a null distribution for change in network potential. Users should be able to compute these network metrics for any mRNA sequencing matrix with little pre-processing required. In addition, the package documentation and structure should facilitate greater transparency of our analysis and allow reviewers and readers to more fairly evaluate our methods. Most of our processed data is now conveniently installed along with the package. We also provide functions for easy remote handling of PPI networks such as biogrid.

As we work in LaTeX, these are not tracked, *per se*, but can be changed back and forth from "blue' (changed) to "black' (clean), by altering line 10 in the code. We have also provided both a "clean" and "tracked" PDF of our manuscript with this re-submission. Many thanks again.

Sincerely,

Davis Weaver and Jacob Scott on behalf of all coauthors

Review response

We will now provide a point by point response to the reviewers comments. Comments from reviewers will be in *italics*, and the subsequent comments in **bolded plain text**.

Reviewer 1

First and foremost, we would like to thank reviewer #1 for their careful assessment of our manuscript.

1. *However, the equation is not balanced from a dimensional perspective. Moles cancels on the RHS so the RHS is pure energy. Whereas, the Gibbs energy has units of energy [J] per mole. So technically, by multiplying by C_i, the authors have a scaling factor of the concentration of protein i. I do not view this as a show stopper, instead I recommend modifying Eq. [1] to indicate that this is a mapping relation.*

   We thank the reviewer for this note. We have edited our manuscript to reflect that our equation was inspired by Gibbs free energy and that we do not expect dimensional concordance (Methods, section 1.4).

Reviewer 2:

We would like to thank reviewer #2 for their careful assessment of our manuscript. Their highly specific and actionable feedback was extremely useful and has led us to substantially improve this work.

1. *...the team has sequenced the transcriptomes of six Ewing's sarcoma cell lines but it is questionable whether the cell lines are representative of patient tumors vs artefacts of cell culturing. There are other RNA-seq studies representing patient tumors that could be used: https://dcc.icgc.org/releases/current/Projects/BOCA-FR, and https://www.ncbi.nlm.nih.gov/pmc/articles/PMC7191197/.*

   This is an excellent point. As the reviewer notes, cell line data often fails to reliably model patient tumors due to the adaptations that tumors take on to survive *in vitro*. To address this, we included consideration of all 15 Ewing sarcoma patient samples provided by St. Jude Children's Research Hospital through the St. Jude Cloud. We described this addition in the methods (section 1.2), as well as the results (sections 2.1, section 2.2, figure 3, and figure 4).

2. *There is no evidence or discussion about the predicted microRNAs being expressed in the tumors of interest. Is there any molecular data to support it? Would the effects of predicted cocktails be attenuated if the microRNAs would be natively expressed in cells and inhibiting the predicted genes already?*

   To address this point, we conducted miRNA sequencing for the 18 Ewing Sarcoma cell line samples under study, and included these data as a heatmap in Figure 5. We also included discussion of these results in the results section (section 2.3).

3. *The authors assume a linear additive effect of multiple miRNAs inhibiting a gene (line 122, page 5/16) and further, use an assumed value of 20% per microRNA. The linear, additive*

relationship is likely problematic if the proposes miRNAs bind similar sequences in the target mRNAs. For example, are the proposed miRNAs LET-7a-3p and LET-7b-3p commonly binding similar mRNAs and target sequences?

We thank the author for this excellent point. We had to make several simplifying assumptions in order to develop our algorithm for the identification of promising miRNA cocktails. We have now included discussion of the potential confounding effects of miRNA that inhibit similar sequences of target mRNAs (discussion section).

4. *The network analysis approach would benefit from a null model that would show if the observed synthetic changes in network potential are truly significant for particular genes or could they be also observed by random chance. To achieve a null model, nodes or edges in the network could be shuffled and potential calculations of the shuffled networks would lead to a baseline distribution used as control.*

   This is extremely useful feedback. To address this point, we implemented a permutation test to determine if changes in network potential that we focused on could be attributed to random chance. We described this permutation test in the methods (section 1.5), and in the results (section 2.2 and figure 3). Of note, this additional piece of work, which we believe significantly strengthens our results, took a fair amount of time to accomplish – and is the reason for the tardiness of our response. Apologies. We do, however, think it was entirely worth it!

5. *The identified genes and microRNAs need to be better described to make the study convincing. What are the network properties of the genes/miRNAs? Are they central or hub-like? Highly or lowly expressed? Known cancer driver genes? etc.*

   We appreciate the need to provide further context for our findings. To address this point, we added a short description of some of these properties to the results section (section 2.2), and added some additional context to the discussion.

Reviewer 3:

We would like to thank Reviewer 3 for their careful reading and thoughtful critique of our manuscript. We will address this reviewer's major comments point-by point below.

Overall, the method is simple and easy to understand. The paper is written in organized fashion and easy to follow with a good command of language. My main concern of this paper is its actual scientific contributions. Without wet lab experiments, simulation experiments, or at least gold-standards, it is impossible to assess how the proposed 3-miRNA cocktail performs in-vitro letting alone in-vivo animal trials. There are many factors that the proposed 3-miRNA cocktail wont work or the methodology itself is questionable.

We agree that for the results presented herein to move to clinical trials, further *in vitro* or even *in vivo* work would need to be performed. However, the concept of this type of combination therapy is brand new, and our network-science based algorithm for determining these cocktails is as well – in addition to the now somewhat comprehensive analysis of data for the rare cancer Ewings sarcoma. Taken together, we feel that these two advances and novel analysis form a significant scientific contribution – especially

given the purview of this journal. While future *in vitro* and *in-vivo* animal trials will surely be needed to translate any predictions made using these methods to the clinic, we strongly believe that these types of experiments are beyond the scope of our current study.

1. *As authors pointed out in the discussion, the PPI network were not derived from cancer cells and it may very well be different from real cancer samples.*

   This is a good point and unfortunately not something that we can address based on the limitations of the available data. The use of the network we have employed is relatively standard in the cancer network/systems biology community.

2. *It is a big leap of faith that the actual patient cancer cells have the RISC machinery to effectively incorporate the 3-miRNA cocktail.*

   While we appreciate that many derangements of normal cellular processes are present in cancer cells, it is our understanding that RISC machinery is ubiquitous to all mammalian cells. Further, substantial evidence from the literature suggests that RISC machinery such as Ago2, SND1 and AEG-1 tend to be overexpressed in cancer cells, reflecting the role of the RISC in silencing tumor suppressor genes [1, 2, 3]. In fact, MOV10, a key component of the RISC, was actually predicted to be a critical component of the cell signaling network of Ewing Sarcoma by our method. We now make note of this key finding in the discussion.

3. *Whats the ideal dosage? Presumably cancer cells can increase the expression of the target genes being repressed by increasing the transcription factors expression, decreasing RISC proteins that are required for the miRNA repression to work, or simply increasing the protein translation*

   This is an excellent point. We made several simplifying assumptions regarding miRNA dosage. We now more clearly note these limitations in the discussion section.

4. *Repressing the genes with the largest network potentials may not necessarily improve the patient outcome. A more realistic approach would be to first look at DE genes between normal and EW patient sample (if any).*

   While this would certainly be an interesting approach, we feel that such an analysis would be beyond the scope of the current study. And, while we can not guarantee our approach will work, we feel that the novel concept and algorithmic method are valuable.

5. *Its completely unclear how to penalize miRNA scores if they do target the housekeeping genes. Currently, it seems that this step of choosing miRNA cocktail is done by mere manual inspection in post-hoc way. So the worst-performing or best-performing miRNAs are the number of house-keeping genes they target.*

   We have updated the methods (section 1.6) to reflect that our approach was in fact *a priori* and based on optimization over a loss function and not manual or *post hoc*. We thank the reviewer for pointing out that this distinction was not made clear in the original text.

6. *When scoring miRNAs, how to avoid redundancy when different miRNAs target the same genes in the 3-miRNA cocktails? For example, if miRNA 1 targets gene A, then the effect of miRNA 2 will not be the same as miRNA 1 if it targets the same gene. This is especially true when the abundance of miRNA 1 is higher than the abundance of gene A.*

   This is an excellent point. We made the simplifying assumption that the effects of additional miRNA would be additive, but this may not be true in all cases. We now make note of this potential limitation in the discussion section.

7. *Why does the approach have to restrict to only the endogenous miRNAs? Can one just design exogeneous ribonucleotide oligos as in the RNA transfection to target desirable target genes?*

   In this study, we limited only to endogenous miRNAs to minimize the well-documented toxicity associated with these miRNA-based therapies. All else being equal, we expect that endogenous miRNAs would have a more favorable toxicity profile compared to exogenous miRNA mimics. We have updated the discussion section to address this important point.

References

[1] Michael Sand, Marina Skrygan, Dimitrios Georgas, Christoph Arenz, Thilo Gambichler, Daniel Sand, Peter Altmeyer, and Falk G. Bechara. Expression levels of the microRNA maturing microprocessor complex component DGCR8 and the RNA-induced silencing complex (RISC) components argonaute-1, argonaute-2, PACT, TARBP1, and TARBP2 in epithelial skin cancer. *Molecular Carcinogenesis*, 51(11):916–922, nov 2012.

[2] Prasanna K. Santhekadur and Divya P. Kumar. RISC assembly and post-transcriptional gene regulation in Hepatocellular Carcinoma, jun 2020.

[3] Sonia A. Melo, Hikaru Sugimoto, Joyce T. O'Connell, Noritoshi Kato, Alberto Villanueva, August Vidal, Le Qiu, Edward Vitkin, Lev T. Perelman, Carlos A. Melo, Anthony Lucci, Cristina Ivan, George A. Calin, and Raghu Kalluri. Cancer Exosomes Perform Cell-Independent MicroRNA Biogenesis and Promote Tumorigenesis. *Cancer Cell*, 26(5):707–721, nov 2014.
