# Supplementary material for: Network potential identifies therapeutic miRNA cocktails in Ewing sarcoma
Source: PLoS Comput Biol. 2021 Oct 18;17(10):e1008755. doi: 10.1371/journal.pcbi.1008755 (PMC8601628; doi:10.1371/journal.pcbi.1008755)
Supplement: Attachment — Submitted filename: rebuttal_letter.pdf [file pcbi.1008755.s009.pdf]

Cleveland Clinic
Department of Translational Hematology and Oncology Research
9500 Euclid Avenue, Cleveland Ohio, 44195, US
September 9, 2021
e-mail: scottj10@ccf.org

Dear Dr Zhaolei Zhang,

We would like to thank all three reviewers and the editor for their second assessment and continued feedback on our manuscript. We are delighted by the positive response from our first round of revisions and have attempted to address the few outstanding concerns in this round of revisions.

As we work in LaTeX, these are not tracked, *per se*, but can be changed back and forth from "blue' (changed) to "black' (clean), by altering line 16 in the code. We have also provided both a "clean" and "tracked" PDF of our manuscript with this re-submission. Many thanks again.

Sincerely,

Davis Weaver and Jacob Scott on behalf of all coauthors

Review response

We will now provide a point by point response to the reviewers comments. We are glad to see that reviewers one and two are satisfied by our first round of revisions and have no further feedback on the content of the paper. In response to the feedback from reviewer 2 regarding the image quality of a few of our figures, We have re-uploaded high quality images for our figures to address this concern. Reviewer 3 was not satisfied with our first round of revisions, and provided specific and actionable feedback about how we could continue to improve our work. We have incorporated these comments into the manuscript, and feel that our work is substantially improved as a result. All code and batch files required to complete these additional analyses are now included in the project github (https://github.com/DavisWeaver/MiR_Combo_Targeting).

Comments from reviewers will be in *italics*, and the subsequent comments in **bolded plain text**.

Reviewer 3:

We would like to thank Reviewer 3 for their careful reading and thoughtful critique of our manuscript. We will address this reviewer's comments point-by point below.

1. *For my comment 1, they said, "The use of the network we have employed is **relatively standard** in the cancer network/systems biology community." I don't think there is such *standard*. It heavily depends on how importantly the PPI network play in the analysis. In this case, the accuracy of the PPI is vital to their prediction.*

   We absolutely agree that the accuracy of the PPI is vital to our predictions. While we feel that biogrid is the most appropriate available resource for protein-protein interactions, we recognize that our results may substantially differ if a different PPI were used. To explore this possibility, we repeated the first phase of our analysis (the selection of drug targets) using the stringdb resource as a sensitivity analysis. We identified broadly similar targets regardless of the PPI used, with the gene set highly enriched in cancer-associated and housekeeping genes. 13 of the top 50 genes were shared between the stringdb and biogrid analyses (see Figure S3). This discrepancy is expected given the importance of the edge-set to our analysis. We also found the distribution of network potential to be very similar between the stringdb and biogrid analyses (see figure S3 and Figure 3).

2. *Also, instead of redirecting the reviewer to a broad section like "We now more clearly note these limitations in the discussion section." to my comment 3 and other comments, the authors should consider directly write their response in the letter for the ease of reviewing. In most cases, I also don't find the relevant revised part in those sections.*

   We apologize for the inconvenience. In the current rebuttal letter, we have attempted to provide the relevant changes directly in the response rather than directing you to the main body of the text

3. *Response to my comment 4 on using real EW patient data for differential analysis is simply begging the question. No effort made in seeking gold-standard or replication cohort/data.*

As we mentioned before, we are unsure what a relevant gold standard would be in this exploratory setting. We certainly appreciate the idea and did think about this suggestion for quite a while. In the end, we felt that expanding our analysis to the patient samples from St. Jude to provide additional context at least partly addressed this. With regards to differential gene expression analysis, we again agree that that is an excellent idea, best implemented in a different study.

4. *Response to my comment 5 says "we have updated the methods (section 1.6) to reflect that our approach was in fact a priori and based on optimization over a loss function and not manual or post hoc.". I can't find the loss function. There is no equation as such.*

   We thank the reviewer for this excellent suggestion, and have now written a loss function in the methods section, lines 144-148 (reperoduced below for ease of review)

   To that end, we designed a loss function (see equation below) to balance the the effects of repressing the housekeeping gene set I as well as the target gene set J:

$$L(\mu) = \sum_{i,j} A(c)(G_i) - A(c)(G_j) \quad \text{for } i \in I \text{ and } j \in J \tag{1}$$

$$A(c) = \begin{cases} 0, & \text{if } c \leq 1 \\ 0.2c, & \text{if } 1 < c < 6 \\ 1, & \text{otherwise} \end{cases} \tag{2}$$

   Where $A(c)$ determines the degree of repression as a function of the number of times, c, that a given gene, i, is targeted by a given miRNA cocktail, μ.

5. *Response to my comment 6 about additive effects is true but also highlight the flaw of the approach.*

   The reviewer is absolutely correct about the additive effects being a significant limitation to our approach. We strongly feel that the work remains useful and interesting despite this limitation.

6. *Figure 3: overall the information content in this figure is low. A scatter plot may be helpful to show that the highly expressed (cancer) genes also have high network potential or maybe there is no correlation.*

   This is an excellent suggestion. We updated figure reworked figure 3 to show a scatterplot with marginal histograms comparing network potential and mRNA expression (sometimes referred to as a jointplot). The original figure (including the patient sample data), has been moved to the supplemental materials.

7. *P5: instead of looking at house-keeping genes and then repeating the analysis without them, why not start the entire analysis without the house-keeping genes in the first place?*

   We had a hard time finding the portion of the paper that this comment referred to. However, I will note that we did not repeat any analyses without housekeeping genes included in the gene set.

8. *P7 line 183: MiR-345-3p or 5p?*

   Thank you for pointing out this error - the miR in question is actually miR-483-3p.

9. *Fig. 5. Panel B does this mean every chosen miRNA still targets 1 house keeping gene? Isnt it still toxic?*

   This is an excellent point - with how promiscous miRNA are, it will be nearly impossible to design any miRNA-based treatment that does not have off-target repression of housekeeping genes. Our hypothesis is that with combination therapy, the doses could be modulated such that significant repression is only observed where the different miRNA in a cocktail overlap.
