# Supplementary material for: Network potential identifies therapeutic miRNA cocktails in Ewing sarcoma
Source: PLoS Comput Biol. 2021 Oct 18;17(10):e1008755. doi: 10.1371/journal.pcbi.1008755 (PMC8601628; doi:10.1371/journal.pcbi.1008755)
Supplement: Attachment — Submitted filename: Weaver etal.docx [file pcbi.1008755.s007.docx]

Weaver et. al.

Ewing sarcoma cocktails

I found the paper most interesting. In particular the concept of ranking the potential targets using a Gibbs energy function – network potential. By first measuring the mRNA of the cell lines and assuming that the mRNA concentration is a surrogate for the protein concentration, the authors used Biogrid PPI (which version? Please state that in the manuscript) and computed the Gibbs function according to their Eq. [1]. However, the “equation” is not balanced from a dimensional perspective. Here is the equation with dimensions in brackets:

$$G_{i}\left[ \frac{J}{mole} \right]\neq c_{i}ln\frac{c_{i}}{\sum_{j=i} c_{j}}[J]$$

Moles cancels on the RHS so the RHS is pure energy. Whereas, the Gibbs energy has units of energy [*J*] per mole. So technically, by multiplying by C_i_, the authors have a scaling factor of the concentration of protein *i*. I do not view this as a “show stopper,” instead I recommend modifying Eq. [1] to indicate that this is a mapping relation.

$$G_{i}\leftarrow c_{i}ln\frac{c_{i}}{\sum_{j=i} c_{j}}$$

Other than that, I applaud the authors for their research and a fine manuscript
